# The development of bispecific antibodies and their applications in tumor immune escape

**DOI:** 10.1186/s40164-017-0072-7

**Published:** 2017-05-02

**Authors:** Xiaolong Zhang, Yuanyuan Yang, Dongmei Fan, Dongsheng Xiong

**Affiliations:** State Key Laboratory of Experimental Hematology, Institute of Hematology and Hospital of Blood Diseases, Chinese Academy of Medical Science and Peking Union Medical College, Tianjin, 300020 People’s Republic of China

**Keywords:** Bispecific antibody, Chemical conjugation, Quadroma, scFv, Cancer immunotherapy, Immune escape

## Abstract

During the past two decades, a great evolution of bispecific antibodies (BsAbs) for therapeutic applications has been made. BsAbs can bind simultaneously two different antigens or epitopes, which leads to a wide range of applications including redirecting T cells or NK cells to tumor cells, blocking two different signaling pathways, dual targeting of different disease mediators, and delivering payloads to targeted sites. Aside from approved catumaxomab (anti-CD3 and anti-EpCAM) and blinatumomab (anti-CD3 and anti-CD19), many more BsAbs are now in various phases of clinical development. Here, this review focus on the development of bispecific antibodies and their applications in tumor immune escape.

## Background

Although considerable evidence supports the hypothesis that immune cells play a vital role in the immune response against cancer, the ability to mount and sustain tumor-specific cellular responses in vivo remains a challenge [1]. Cancer immunotherapy, which harnesses the immune system to battle cancer [2], was named “2013’s Breakthrough of the Year” by *Science* [3]. Cancer immunotherapy was proposed decades ago but has only recently been realized as a promising approach to cancer treatment due to the success of immunomodulating anti-CTLA-4 and anti-PD-1 monoclonal antibodies against various of cancers [4].

In addition to immunomodulating antibodies, bispecific antibodies (BsAbs) are another promising strategy to fight cancer by directly redirecting immune cells to tumor cells. BsAbs have a long history [5], starting in the 1960s when antigen-binding fragments (Fabs) from two different polyclonal sera were re-associated into bispecific F(ab’)_2_ molecules [6]. A bispecific antibody is based on a conventional monoclonal antibody, and it can recognize and bind two different antigens or epitopes simultaneously. Thus, BsAbs show several advantages [1, 7–9]: (1) BsAbs can redirect specific immune effector cells to the proximity tumor cells to enhance tumor killing, which is not achievable with a combination monoclonal antibody strategy; (2) BsAbs can potentially increase binding specificity by interacting with two different cell-surface antigens instead of one; (3) BsAbs offer an opportunity to reduce cost in terms of development, production clinical trials, and regulatory reviews, compared to the single antibody-based agents development in combination therapies; (4) BsAbs will enable the simultaneous blocking of two different pathways that exert unique or overlapping functions in pathogenesis.

The development of BsAbs has long been hampered due to manufacturing problems such as product instability, low expression yields, and immunogenicity [10]. With the development of molecular cloning technology and antibody engineering, there are diverse bispecific antibody formats from which to choose to pursue the optimal biological activity and clinical purpose [11]. There are around 100 different bispecific antibody formats, including small molecules solely of the antigen-binding sites of two antibodies, molecules with an IgG format, and large complex molecules composed of different antigen-binding moieties usually combined with dimerization modules [9]. The engineering of monospecific antibodies to be bispecific opens up a variety of potential therapeutic applications as evidenced by the more than 30 BsAbs currently in clinical development [12]. And the BsAbs against cancers in clinical development were summarized in Table 1.

**Table 1:** BsAbs against cancers in clinical development

Molecule	Targets	Format	MOA^a^	Indication	Status^b^	Developed by
Catumaxomab	CD3 + EpCAM	TrioMab	T cell recruitment	Malignant ascites Gastric cancer Ovary cancer Epithelial cancer	Market 2 2 1-2	Fresenius Biotech
FBTA05	CD3 + CD20	TrioMab	T cell recruitment	BCL	1-2	Fresenius Biotech
Ertumaxomab	CD3 + Her2	TrioMab	T cell recruitment	Metastatic breast cancer	2	Fresenius Biotech
Blinatumomab (MT103)	CD3 + CD19	BiTE	T cell recruitment	B-ALL Relapsed/refractory ALL Pediatric ALL Relapsed NHL	Market 2 1–2 1	Amgen
MT110	CD3 + EpCAM	BiTE	T cell recruitment	Colorectal cancer Lung and gastrointestinal cancer	1 1	Amgen
MT111	CD3 + CEA	BiTE	T cell recruitment	Gastric cancer advanced adenocarcinoma	1b	Amgen
AMG330	CD3 + CD33	BiTE	T cell recruitment	Relapsed/refractory AML	1	Amgen
MT112	CD3 + PSMA	BiTE	T cell recruitment	Prostate cancer	1	Bayer
RG7221	Angiopoietin 2 + VEGF	CrossMab	Two-ligand inactivation	Colorectal cancer	2	Roche
RG7597	Her1 + Her3	DAF-IgG	Two-RTK inactivation	Head and neck cancer, colorectal cancer	2	Genentech
MM111	Her2 + Her3	scFv-HSA	Two-RTK inactivation	Advanced gastric and esophageal cancer	2	Merrimack
MM141	IGF1R + Her3	scFv-IgG	Two-RTK inactivation	Advanced solid tumors	1	Merrimack
MGD006	CD3 + CD123	DART	T cell recruitment	AML	1	Macrogenics and Servier
MGD007	CD3 + GPA33	DART-Fc	T cell recruitment	Colorectal cancer	1	Macrogenics and Servier
AFM11	CD3 + CD19	TandAb	T cell recruitment	Non-Hodgkin’s lymphoma	1	Affimed
AFM13	CD30 + CD16	TandAb	NK cell recruitment	Hodgkin’s disease	1	Affimed
LY3164530	Her1 + cMET	orthoFab-IgG	Two-RTK inactivation	Solid tumors	1	Eli Lilly
TF2	CEA + hapten	D&L Fab3	Payload delivery	Colorectal cancer	1	Immunomedics

Information from ClinicalTrials.gov (http://clinicaltrials.gov)

^a^MOA, mode of action

^b^1, phase 1 clinical trials; 2, phase 2 clinical trials

Like armed monoclonal antibodies, BsAbs do not occur naturally in human body and must be produced by either recombination DNA or cell-fusion technologies. And BsAbs are mainly produced by three methods [13]: (1) chemical conjugation, which involves chemical cross-linkers; (2) quadroma technology based on the somatic fusion of two different hybridoma cell lines; (3) genetic approaches using recombinant DNA technology. This review focuses on the development of the strategies to generate recombinant bispecific antibodies and strategies to reverse immune escape in the treatments.

### Generation of BsAbs

#### Chemical engineering of BsAbs

Chemical conjugation of two different purified monoclonal antibodies was employed to create BsAbs by oxidative recombination firstly in 1961 [6]. Two purified monoclonal antibodies were conjugated through a cross-linker such as the bispecific antibody anti-CD3 × anti-GD2 (3F8BiAb) which was designed to redirect activated T cells to GD2-positive neuroblastomas [14]. Alternative approach is to yield Fab fragments through enzymatic digestion and reduction of desired specific purified antibodies. Bifunctional reagents, which bind to the Fab fragments, are then added to allow for heterodimer assembly by association of the Fab fragments.

However, it is difficult to purify the bispecific heterodimers from homodimers because of the heterogeneity of the end products. And another drawback of chemical cross-linking is poor stability and decreased activity of the antibodies. To improve the purity and yield of products, a scalable method to prepare BsAbs, which was named controlled Fab-arm exchange (cFAE), was developed [15, 16]. The process involves separate expression of two parental antibodies, each containing single matched point mutations in the CH3 domains (F405L and K409R, respectively). Then the parental antibodies (IgG1-F405L-EGFR and IgG1-K409R-CD20) are mixed and subjected to controlled reducing conditions (incubated with 50 Mm 2-mercaptoethylamine-HCl for 5 h at ambient temperature) in vitro that separate the antibodies in HL half-molecules and allow reassembly and re-oxidation to form highly pure BsAbs. And this process results in generating BsAbs with a greater than 90% heterodimerization efficiency and greater than 90% yield [16]. Additionally, Suparna Paul et al. proved that the process could be accelerated by conducting the cFAE using culture supernatants from separate cultures that were producing the respective parental antibodies with matched mutations prior to purification [17], which provides an shorter processing time with potential benefits in large-scale BsAbs preparation.

#### Quadroma technology for BsAbs

Another early attempt to produce BsAbs employed quadroma technology. This approach is based on the somatic fusion of two different hybridoma cells producing monoclonal antibodies with the desired specificity. BsAbs produced by quadromas resemble conventional antibodies, which retain Fc-mediated effector functions such as antibody-dependent cell-mediated cytotoxicity (ADCC), complement-dependent cytotoxicity (CDC), and antibody-dependent cellular phagocytosis (ADCP) [10]. Additionally, the Fc region of BsAbs favor purification and improves solubility and stability. And because of their large size and FcRn-mediated recycling, BsAbs with IgG-like formats usually have longer serum half-lives [12].

Because quadromas express two different immunoglobulin heavy and light chains that assemble randomly, nonfunctional antibodies are also produced. However, mispaired by-products could be significantly decreased by fusing two hybridomas of different species such as mouse/rat quadroma. Catumaxomab, produced by fusion of a mouse hybridoma and rat hybridoma, is the first approved bispecific antibody in 2009 for the treatment of malignant ascites in patients with EpCAM-positive Tumors [18]. Interestingly, the resulting hybrid mouse/rat Fc portion efficiently interacted with activating human Fc receptors (FcγRI and FcγRIII), but not inhibitory ones (FcγRIIB) [19]. However, catumaxomab is a mouse-rat hybrid IgG molecule, human anti-mouse or anti-rat antibody response are observed in most patients [12]. Several other BsAbs have been produced using similar quadroma technology. For example, FBTA05 [20] (anti-CD3 and anti-CD20) and ertumaxomab [21] (anti-CD3 and anti-HER2) are in different phases of clinical development for patients with relapsed or refractory B-cell lymphoma or metastatic breast cancers, respectively.

Homodimerization of the two heavy chains of IgG is mediated by the interaction between CH3 domains. To overcome the problem of undesirable heavy-chain pairing, a strategy called “knobs-into-holes” [22] was developed. The knobs-into-holes scenario employs a “knob” mutation (T366W) and pairing “hole” mutations (T336S, L368A, Y407V) in the CH3 domains [23]. In this approach a “knob” variant was obtained by replacement of a small amino acid with a larger one in the CH3 domain, which was desired to insert into a “hole” in another CH3 domain created by replacement of a large residue with a small one [23]. The mutated CH3 domains could facilitate heterodimerization of heavy chains [23]. However, another challenge for BsAbs in this format is the problem with light chain mispairing. To circumvent this, several methods have been proposed [24]: (1) generating BsAbs with common light chains; (2) expressing the knob- and hole-containing half-molecules separately in different bacteria; (3) combining CrossMab and knobs-into-holes strategies; (4) introducing additional mutations into VH–VL and CH1–CL interfaces.

#### Genetic engineering of BsAbs

By using molecular cloning technology, BsAbs can be constructed with some or all of the constant domains of an antibody. BsAbs in this group can be divided into two categories: IgG-like formats and non-IgG-like formats. As mentioned above, IgG-like formats are BsAbs bearing an Fc region, which retain Fc-mediated effector functions. These formats roughly include “knob into hole” IgG, crossMab, ortho-Fab IgG, DVD-Ig, two in one IgG, IgG-scFv and scFv_2_-Fc [12].

Here, we focus on the non-IgG-like formats of BsAbs. The smaller size of such antibodies offers a better tumor tissue penetration over IgG-like formats. In this format, the variable domains of each parental monoclonal antibody and the linkers are cloned and linked to form a single-chain bispecific antibody. These bispecific antibodies represent many formats, including tandem scFvs, diabody format, single-chain diabodies, tandem diabodies (TandAbs), dual-affinity retargeting molecules (DARTs), dock-and-lock (DNL), and nanobodies [24].

Among these bispecific antibody constructs, tandem scFvs and diabodies have been intensively studied. Tandem scFvs are two scFv fragments linked by an extra peptide linker such as glycine-serine repeat motifs [13]. The most frequently-used domain order is VL_A_-linker1–VH_A_-linker2–VH_B_-linker3–VL_B_ (VL and VH derive from the single chain antibody fragment; A and B represent the parental monoclonal antibody A and B) [25]. The length of the linker1 and linker3 determines the polymerization situation of scFv, while the linker2 determines the movement flexibility between two scFvs. The short linker prevents intra-chain but not inter-chain pairing of the VL and VH domains. And the long flexible linker permits antigen-binding sites to rotate freely. Like one of the best known bispecific T cells engagers (BiTEs) blinatumonmab, two longer linkers are placed between the light chain and heavy chain, and a short linker is used to bridge the two scFvs in tandem format [26]. Although it is difficult to be produced in E. *coli*, BiTEs are well-expressed in mammalian cells. In the diabody format, the VH of the first antibody is linked to the VL of the second antibody, while the VL of the first antibody is linked to the VH of the second antibody. In addition, this format has been improved by adding an inter-chain disulfide bond between the two polypeptides in order to decrease the amount and stability of homodimers, which were called DARTs [27].

Compared with IgG-like formats, scFv-based BsAbs have many advantages including less immunogenic, ease of manufacturing, and enhanced tissue penetration. However, their short half-lives due to their small sizes and lack of Fc region become a drawback in the clinical applications. For example, blinatumomab is administrated over a 28-day continuous infusion using a mini-pump in order to maintain a steady drug concentration [28], which results in inconvenience for patients and an increased possibility of treatment-related adverse event. To overcome this drawback, a tetravalent TandAbs format is developed. TandAbs contain two pairs of VL and VH domains connected in a single polypeptide chain [29]. Upon expression, two polypeptide products dimerize in a head-to-tail fashion, forming homodimers with large molecular weight (~105 kDa) [30]. AFM11 is a tetravalent bispecific TandAb targeting CD19 and CD3 with a half-life ranging from 18.4 to 22.9 h after intravenous administration in mice [29]. Because of its excellent preclinical outcomes, AFM11 is entering clinical trials and currently recruiting patients with relapsed and/or refractory CD19 positive B-cell NHL (ClinicalTrials.gov: NCT02106091) and patients with relapsed or refractory adult B-precursor ALL (ClinicalTrials.gov: NCT02848911). Another TandAb AFM13 (anti-CD30/anti-CD16A) is designed for the treatment of CD30-positive malignant lymphoma by redirecting and activating NK cells. AFM13 is now entering phase II clinical trials in patients with relapsed of refractory Hodgkin Lymphoma (ClinicalTrials.gov: NCT02321592).

In addition to multimerization, the serum half-life of single-chain-based BsAbs can be extended by other available strategies including conjugating a single polyethylene glycol (PEG) chain [31], fusion with human serum albumin (HSA) [32], or fusion with an Fc fragment [33].

### BsAbs in tumor immune escape

A CD33/CD3-bispecific BiTE construct called AMG330 has been designed to target acute myeloid leukemia (AML) [34]. In preclinical studies, AMG330 was shown to be very effective in recruiting and activating autologous T cells [34]. However, reduced T-cell activation and decreased tumor cell lysis was observed in some patient cases [35]. Recently, a study from C. Krupka, et al. [36] showed that although PD-1 and PD-L1 were not expressed at a relevant level at time of diagnosis, their expression was induced by AMG330-mediated T-cell activation in primary AML patient samples. They also demonstrated that blockade of the PD-1/PD-L1 interaction augmented lysis of AML cells by AMG330. Thus, the use of bispecific antibodies especially which leads to strong T-cell activation and production of proinflammatory cytokines might also trigger tumor cells to employ immunosuppressive strategies to escape antibody-mediated tumor cell lysis.

Aside from the PD-1/PD-L1 axis, the CD47/SIRPα interaction should also be noted. CD47 is a ubiquitously expressed immune checkpoint receptor that is usually up-regulated in cancers [37]. CD47 could interact with its counter-receptor SIRPα on macrophages and other myeloid cells to inhibit tumor cell phagocytosis and trigger immune evasion [38, 39]. For this reason, IgG-based bispecific antibodies were created to block CD47 combining tumor targeting. These bispecific antibody constructs including anti-CD47/CD20 [40], anti-CD47/CD19 [41], and anti-CD47/MSLN [41]. By neutralizing CD47, tumor cells could be efficiently killed effector cells mediated by their Fc portion.

As a subclass of growth factor receptors, receptor tyrosine kinases (RTKs) play a vital role in oncogenesis [42]. Although several monospecific antibodies targeted RTKs have been approved for cancer treatment, simultaneously blocking two RTKs with BsAbs may offer better therapeutic potential than monoclonal antibodies. The Her family of RTKs has four members, HER1/EGFR, HER2, HER3, and HER4. HER2-HER3 heterodimerization leads to breast cancer cell proliferation and is involved in transformation [43]. Then MM-111, a BsAb with two scFv fused to modified HSA in phase 2 study, was developed to bind to both of HER2 and HER3 [44]. Another BsAb in phase 1 study is MM-141, which bind HER3 as well as insulin-like growth factor-1R (IGF1R) with a scFv-IgG format. Thus, simultaneous inhibition of two RTKs could be a valid strategy to overcome escape of tumors. And several other BsAbs that inhibit two RTKs are included in Table 1, such as RG7597 and LY3164530.

On the other hand, tumor cells may down-regulate the antibody’s target antigen and escape recognition during the treatment, which is another major escape mechanism. Multiple clinical trials have shown that anti-CD19 chimeric antigen receptor T cells (CART19) have curative potential against relapsed B-cell malignancies [45]. However, a recent trial of CD19 CAR T-cell therapy revealed that 90% of patients acquired a complete response, but 11% of those patients eventually relapsed with CD19-negative tumors [46]. The probability of antigen escape by spontaneous mutation and selective expansion of antigen-negative tumor cells decreases with each additional antigen that can be recognized by the CAR T cells. Therefore, a potential strategy against antigen escape is to combine bispecific antibodies to generate T cells that could recognize multiple antigens. The first bispecific CAR T cells, which could simultaneously recognize both of CD19 and CD20, were developed to prevent antigen escape by malignant B cells [47].

## Conclusions

As the next generation of strategies for cancer therapy, bispecific antibodies have acquired much attention owing to their unique mechanism of action. Although just only two of BsAbs have gained marketing approval, numerous designed BsAbs are now being tested in clinical trials [24] not only for cancers, but also for other diseases.

Future advances in BsAbs technology will be focused on the development of new platforms which encompass the entire process from discovery and preclinical studies to clinical material production. On the other hand, the discovery of new targets is also urgently needed to increase efficacy and reduce adverse effects of bispecific antibodies. The complexity of tumors should also be in consideration during the process of cancer therapy. Thus, bispecific antibodies might probably be combined with other therapeutics such as checkpoint antibodies, IDO inhibitors, or vaccines.

In conclusion, new formats and producing methods of bispecific antibodies should be persistently developed. And continued persistence is needed in the anti-cancer battle.

## Abbreviations

ADCC: antibody-dependent cell-mediated cytotoxicity
ADCP: antibody-dependent cellular phagocytosis
AML: acute myeloid leukemia
ALL: acute lymphoblastic leukemia
BCL: B cell lymphoma
BiTE: bispecific T cell engager
BsAbs: bispecific antibodies
CAR: chimeric antigen receptor
CDC: complement-dependent cytotoxicity
cFAE: controlled Fab-arm exchange
DARTs: dual-affinity retargeting molecules
DNL: dock-and-lock
Fab: antigen-binding fragment
HSA: human serum albumin
NHL: non-Hodgkin’s lymphoma
NK: natural killer cells
PEG: polyethylene glycol
RTK: receptor tyrosine kinase
scFv: single- chain variable fragment
